# Toxicity of a novel antifungal agent (ATB1651 gel) in Yucatan minipigs *(Sus scrofa)* following 4 weeks of daily dermal administration

**DOI:** 10.1007/s43188-023-00222-z

**Published:** 2024-02-06

**Authors:** Hyung-Sun Kim, Goo-Hwa Kang, Mi-Jin Yang, Yun-Jeong Joo, Dong-Gi Lee, Han-Seung Lee, Jong-Seung Lee, Jeong Ho Hwang

**Affiliations:** 1 Animal Model Research Group, Jeonbuk Branch Institute, Korea Institute of Toxicology, Jeongup, Jeonbuk 56212 Republic of Korea; 2 Jeonbuk Pathology Research Group, Jeonbuk Branch Institute, Korea Institute of Toxicology, Jeongup, Jeonbuk 56212 Republic of Korea; 3 Jeonbuk Quality Assurance Unit, Jeonbuk Branch Institute, Korea Institute of Toxicology, Jeongup, Jeonbuk 56212 Republic of Korea; 4 AmtixBio Co., Ltd., Hanam-si, Gyeonggi-do 12925 Republic of Korea

**Keywords:** Antifungal agent, Minipig, No-observed-adverse-effect level, 4-week repeated-dose toxicity

## Abstract

ATB1651 gel is an antifungal drug candidate that enhances antifungal activity through substitution of several aryl rings, alkyl chains, and methyl groups. To ensure safety of use of ATB1651 gel, assessment of its potentially toxic side effects is necessary. In this study, we examined the repeated-dose toxicity of ATB1651 gel to Yucatan minipigs *(Sus scrofa)* in accordance with the Good Laboratory Practice guidelines. Five doses of ATB1651 gel (0%, 0.2%, 0.5%, 1.0%, 3.0%) were administered dermally to the left and right flanks of 38 minipigs daily for 4 weeks. Mortality, clinical symptoms, dermal scores, body weights, and physiological, biochemical, pathological, and toxicokinetic analyses were performed after the treatment period. No systemic toxicological damage was observed in either male or female minipigs regardless of dose; however, dermal application of ATB1651 gel caused some skin alterations at the application sites. Specifically, erythema and eschar formation, edema, and scabs or raise spots were observed at the application site(s) in males in the 3.0% ATB1651 gel treatment group and in females at ATB1651 gel concentrations ≥ 1.0%, with dermal scores ranging from grade 1 to 2. Additionally, histopathological assay indicated infiltration of different types of inflammatory cells and the presence of pustule/crust at the application site(s) in both males and females at ATB1651 gel concentrations ≥ 0.5%. However, these changes were reversible after a 2-week recovery period and were considered a local irritation effect of ATB1651 gel. The no-observed-adverse-effect level of ATB1651 gel was 3.0% with regard to topical and systemic toxicity in both male and female minipigs. Collectively, our results imply that ATB1651 gel is a safe candidate for clinical development as an antifungal drug with a wide therapeutic window.

## Introduction

According to the Centers for Disease Control and Prevention (CDC), antifungal agents exert their effects against fungal infections by killing or stopping the growth of fungi [1]. Several types of antifungal agents have been developed, including polyenes, azoles, allylamines, echinocandins, and pyrimidines that target the cell membrane directly or indirectly depending on the mode of action [2–4]. The first introduced antifungals were polyenes, such as amphotericin B, nystatin, and natamycin, which are still in use since their discovery in the 1950s [5]. However, polyenes are known to cause renal and hepatic toxicities owing to their affinity for human cholesterol [6]; anemia, neutropenia, or thrombocytopenia have been observed in some patients [7]. Azoles were first introduced in the 1960s [5], and have been used as fungicides in humans and animals and biocides in vegetables and fruits due to their high stability and broad spectrum. Additionally, azoles are used in various fields as an anti-icing liquids and corrosion inhibitors [7, 8]. However, azoles have been reported to cause cardiac issues [9] and hepatotoxicity [10–13]. Allylamines became commercially available in the 1990s [5], including terbinafine, which has been reported to be nephrotoxic [14], gastrointestinal tract toxic, and skin-toxic. Moreover, rare hepatotoxicity, neutropenia, and toxic epidermal necrolysis have been reported in humans [15].

To ensure high efficacy, researchers have developed fungicides with different routes of administration, among which topical and oral forms are the most widely used [16]. Most of the toxicities mentioned above are associated with oral administration; thus, topical antifungal drugs are preferred for preventing systemic toxicity. One of the toxicological tests employed to assess the safety of a substance is acute dermal toxicity, which determines if a substance can cause irreversible damage to the skin and/or systemic toxicity after dermal application [17]. Commonly, rodents and rabbits are used for dermal toxicity studies; however, the use of minipigs has increased recently owing to similarities with humans in the anatomy and physiology of their skin [18–24]. Additionally, regulatory agencies such as the U.S. Food and Drug Administration (FDA) and Organization for Economic Co-operation and Development (OECD) have encouraged the use of minipigs in dermal toxicity studies [18].

For the treatment of onychomycosis and dermatomycosis, ATB1651 gel is a promising antifungal agent. A previous study showed that ATB1651 inhibited fungal growth by activating cell-wall integrity-related pathways and disrupting fungal integrity [3]. However, the potential toxicity of ATB1651 gel is yet to be examined. Here, we investigated the potential subacute toxicity of ATB1651 in minipigs following daily dermal application for 4 weeks and assessed the reversibility of any toxic effects during a 2-week recovery period.

## Materials and methods

### Antifungal agents

The test substance used in this study was ATB1651 gel, a prospective antifungal agent produced by MedPharm Ltd., a Good Manufacturing Practice (GMP) facility in the UK. Preparation analyses including stability, homogeneity, and concentration analyses were also conducted.

### Test guidelines for dermal toxicity study

Dermal administration of ATB1651 gel to minipigs (Yucatan) was performed at the Contract Research Organization (CRO). This study was conducted in compliance with Good Laboratory Practice (GLP) regulations, the Ministry of Food and Drug Safety Notification No. 2017–71 Test Guidelines for Safety Evaluation of Drugs and Annex 2 Repeated Dose Toxicity Study, and the ICH Harmonized Tripartite Guidelines M3 (R2) Guidance on Nonclinical Safety Studies for the Conduct of Human Clinical Trials and Marketing Authorization for Pharmaceuticals.

### Animals

All experimental procedures were approved by the Institutional Animal Care and Use Committee of the Korea Institute of Toxicology (KIT-2112-0092). Animal management and care were in accordance with the guidelines of the Association for the Assessment and Accreditation of Laboratory Animal Care (AAALAC). Yucatan minipigs (*Sus scrofa*) weighing 10–12 kg (3–5 months old) were housed individually in stainless-steel slide bottom cages during the study period to avoid cross-contamination of the test items and application sites. The animal house was maintained under the following controlled conditions: temperature, 19–27 °C; relative humidity, 30–70%; ventilation, 10–20 times per hour; and ~ 12-h light cycle at 300–700 lx. The minipigs were fed gamma irradiation-sterilized diets (~ 300 g per head) once daily.

### Determination of body surface area (BSA)

The dosing site was determined as 10% of body surface area (BSA) according to the OECD test guideline No. 410. First, total body surface was determined using the following formula: BS (m^2^) = (70 × BW^0.75^)/1000 for pigs weighing 6–30 kg [25], where BW was the maximum body weight for each sex. The 10% BSA was recalculated based on the recent body weight measured once per week. To avoid the spine, the 10% BSA was split into 2 areas: 5% BSA on the left and 5% BSA on the right side of the spine, with ~ 4 cm distance between both application sites. Prior to dermal application, hair at the dosing sites was clipped or shaved using a clipper, and each corner of the dosing site was tattooed. The procedures were performed under sedation using ketamine and xylazine. During the administration period, hair at the administration site was gently removed to maximize ATB1651 gel absorption.

### Dermal administration

The experimental period comprised 7–8 days of pretreatment to obtain initial data, a 4-week treatment period, and a 2-week recovery period. Yucatan minipigs (n = 38) were randomly assigned to 1 control group (0% ATB1651 gel) and 4 treatment groups (0.2, 0.5, 1.0, and 3.0% ATB1651 gel). Each group comprised 3 males and females except the control and 3.0% groups, which contained an additional 2 males and 2 females to evaluate reversibility during the recovery period. In a previous 4-week dermal administration study of liquid formulation in minipigs, the systemic no-observed-adverse-effect level (NOAEL) was determined at 2.0%. It was considered that the turns from liquid to gel type of the test item formulation due to the change in vehicle control item would weaken dermal toxicity, so the high dose level was determined to be 3.0% (1.0% higher than in the previous study). Therefore, the high-dose level used in the present study was 3.0%, the middle-dose levels were 1.0% and 0.5%, and the low-dose level was 0.2% (no expected toxicity).

Both control and test formulations were gently and uniformly applied to the open application sites (10% BSA) and administered dermally once daily for 4 weeks (dose volume of at least 5 μL/cm^2^) using a soft spatula. Administration was performed in the cages without animal restraint. The exposure time for the control and test formulations was ~ 6 h; application sites were subsequently rinsed with clean water to remove any residual formulations and dried with porous gauze. The administration site was not occluded with gauze during formulation exposure, as the formulation absorption time was short (as determined through our own preliminary tests).

### Observation and measurements

Most experimental measurements and observations, including body weight, food consumption, clinical signs, ophthalmic examination, electrocardiogram, and dermal scoring, were recorded using Pristima XD software (Version 7.4. Xybion Corporation; Lawrenceville, New Jersey, USA).

Mortality and clinical signs, such as moribund state, general appearance, and behavioral changes, were observed once daily during the entire experimental period and twice daily during the treatment period. Body weight and food consumption were measured weekly during the entire study period. Ophthalmological and electrocardiographic examinations were conducted by a veterinarian under sedation with ketamine (11–12 mg/kg) and xylazine (2–3 mg/kg) for all live animals during pretreatment, test treatment (day 24), and the recovery period (day 9 or 10). A slit lamp (XL-1, Ohira Co., Ltd., Japan) and binocular indirect ophthalmoscope (Vantage Plus Digital, Keeler Ltd., England) were used for ophthalmic examination of animals after receiving 1–2 drops of a mydriatic agent (Mydriacyl Oph Soln 1%, Alcon, Geneva, Swiss) to both eyes. Cardiac electrocardiogram intervals (QT, QTc, PR, and QRS) and heart rate were measured using an electrocardiograph (Cardio XP; Bionet Co., Ltd., Korea). QTc at each time point was obtained using Bazett’s formula [26, 27]. During treatment and recovery periods, the administration site of each minipig was observed in detail once a week before dosing for erythema, edema, and eschar formation. Dermal scores were recorded according to Draize [28].

### Clinical pathology examination

Blood obtained from the jugular vein or vena cava was collected in EDTA-2K tubes, 3.2% sodium citrate tubes, and serum-separating tubes to assess hematology, coagulation, and clinical chemistry, respectively. Clinical pathology examination was performed 2–3 times during the entire study period on the following days: one week before administration, day 29 for all animals, or day 43 for recovery animals. All animals were fasted for ~ 16 h before blood sampling and provided with water ad libitum.

Hematological parameters, including total white blood cell (WBC) count, total red blood cell (RBC) count, hemoglobin, hematocrit, mean corpuscular volume, mean corpuscular hemoglobin, mean corpuscular hemoglobin concentration, platelet count, reticulocyte count absolute, reticulocyte count relative, neutrophil count absolute, neutrophil count relative, eosinophil count absolute, eosinophil count relative, basophil count absolute, basophil count relative, monocyte count absolute, monocyte count relative, lymphocytes absolute, lymphocytes relative, large unstained cells absolute, and large unstained cells relative, were measured using an ADVIA2120i hematology analyzer (Siemens, USA). Coagulation parameters, including prothrombin time and activated partial thromboplastin time, were measured using an ACL Elite Pro coagulation analyzer (Instrumentation Laboratory, Italy). Clinical parameters, including glucose, BUN, creatinine, total protein, albumin, albumin/globulin ratio, total cholesterol, triglyceride, phospholipid, AST, ALT, total bilirubin, AP, gamma-glutamyl transpeptidase, creatine phosphokinase, calcium, inorganic phosphorus, sodium, potassium, and chloride, were measured using a TBA 120FR chemistry analyzer (Toshiba Co., Japan).

Urine was collected from the cage pans of all animals on the day before administration, terminal sacrifice (day 29), and recovery sacrifice (day 43) for analysis. Before urine collection, the animals were fasted overnight; however, drinking water was provided. Urine volume was recorded using a measuring cylinder, and the following parameters were measured using a Cobas U411 urine analyzer (Roche, Switzerland) with a urine reagent strip: color, clarity, pH, specific gravity, bilirubin, protein, urobilinogen level, nitrite level, glucose level, erythrocyte count, ketone, leukocyte count, urine potassium, urine chloride, urine sodium, urine cast, epithelial cell count, WBC count, and RBC count.

### Necropsy and histopathological examination

All animals were anesthetized intravenously via the ear vein using thiopental sodium (75–80 mg/kg) after intramuscular sedation with ketamine (11–12 mg/kg) and xylazine (2–3 mg/kg) on the day of terminal sacrifice (day 29) and recovery sacrifice (day 43), and then euthanized by exsanguination. Tissue samples collected from all animals at the terminal and recovery sacrifices were examined macroscopically by a veterinary pathologist. The brain, adrenal glands, pituitary gland, testes, liver with gall bladder, epididymides, spleen, lung with bronchi, heart, thyroid, thymus, uterus and cervix, salivary glands, ovaries with oviducts, seminal vesicle, prostate, and kidneys were weighed. All organs were preserved in 10% neutral buffered formalin, except the eyes (with optic nerve), which were fixed in Davidson’s fixative and the testes and epididymides, which were fixed in Bouin’s fixative for ~ 24 to 72 h and transferred to 70% ethanol. Formalin was infused into the lungs via the trachea and urinary bladder. Subsequently, the sections were embedded in paraffin, sectioned (section thickness: 2.5 μm), stained with hematoxylin and eosin (H&E), and examined under a microscope (BX53, Olympus, Tokyo, Japan). Images were obtained at × 200 magnification using a microscope and evaluated microscopically by a veterinary pathologist. The following tissues were examined and analyzed in this study: abnormal lesions, adrenal glands, animal ID, aorta (thoracic), brain, cecum, colon, duodenum, epididymis, esophagus, eyes with optic nerve, femur with marrow, heart, ileum, jejunum, kidneys, liver with gall bladder, lung with bronchi, mammary gland, uterus with cervix, vagina, injection sites, pancreas, prostate, pituitary gland, rectum, salivary glands, sciatic nerve, seminal vesicles, skeletal muscles, skin, thoracic spinal cord, spleen, sternum with marrow, stomach, testes, thymus, thyroids, tongue, trachea, urinary bladder, mesenteric lymph node, ovaries, and mandibular lymph nodes.

### Statistical analysis

All data were analyzed using Prism (Version 8; GraphPad Software, San Diego, CA, USA) and Pristima software (Version 7.4; Xybion Medical Systems Corporation, Lawrenceville, New Jersey, USA). Data were analyzed for homogeneity of variance using Bartlett’s test. Homogeneous data were analyzed using analysis of variance (ANOVA), followed by Dunnett’s test for multiple mean comparison. Heterogeneous data were analyzed using a Kruskal–Wallis test, followed by Dunn’s rank sum test to compare the control and experimental groups. After performing an F test to assess homogeneity of variance between the control and recovery groups, a Student’s *t* test was conducted to analyze significant differences between the homogeneous data groups. The Wilcoxon rank-sum test was used to assess differences between the heterogeneous data groups. Statistical significance was set at *p* < 0.05.

## Results

### Observations and measurements before necropsy

#### Formulation analysis

ATB1651 gel formulations in the range of 0.2–3.0% have been shown to be stable for 2 years under storage at room temperature in the dark. The formulations exhibited homogeneous distribution at low, middle, and high ATB1651 gel concentrations on day 1 and week 4, with coefficients of variation (CV) within 5% (95–105%). Concentration analysis showed stability within 15% CV (101.6–102.9% and 97.0–102.7% on day 1 and week 4, respectively.

#### Body weight

**Fig. 1:**
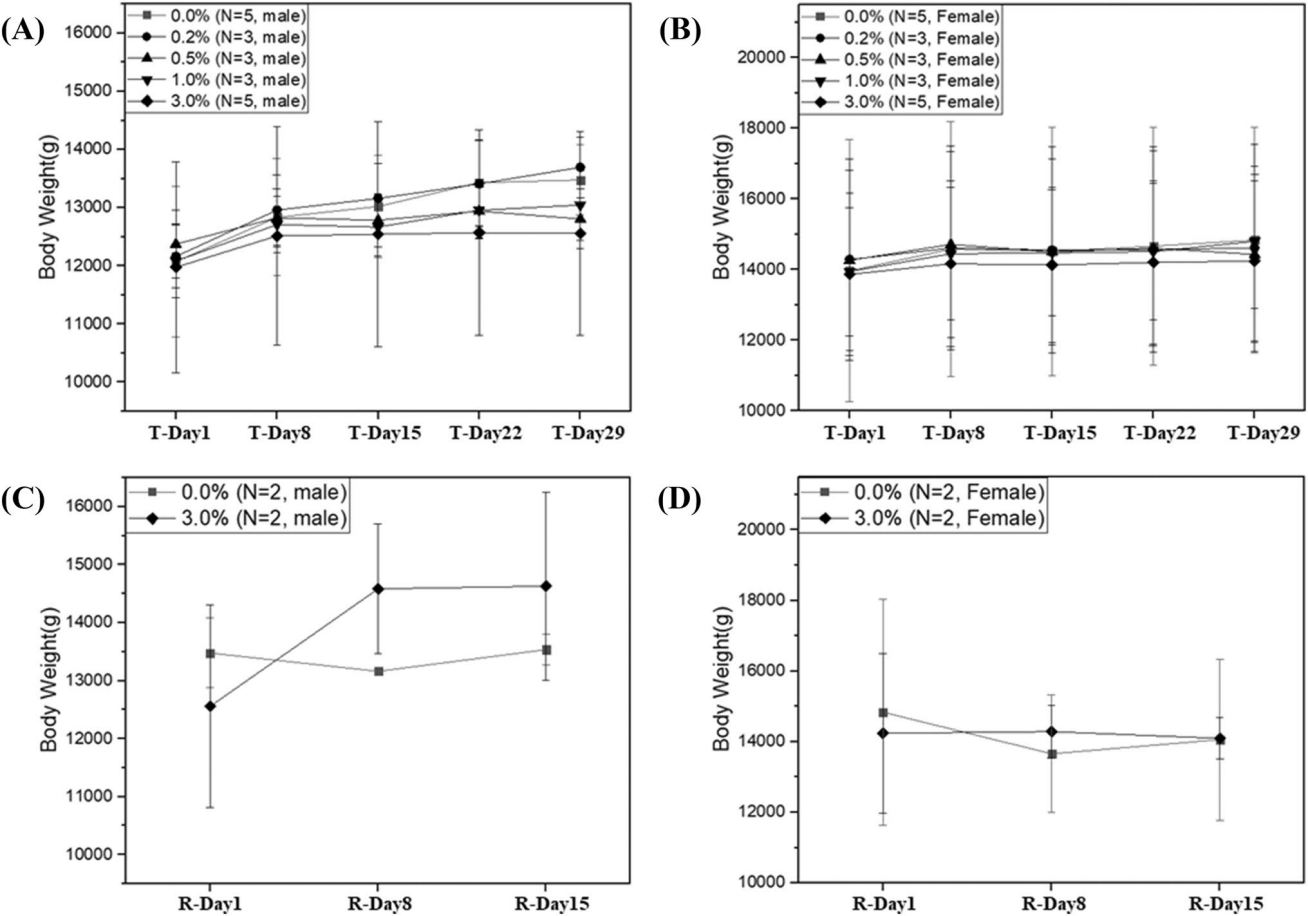
Graph of body weight (mean ± standard deviation) of minipigs receiving a 4-week-repeated-dose of ATB1651 gel via dermal administration. Mean body weight changes in males (**a**) and females (**b**) of all groups during the treatment period, and in males (**c**) and females (**d**) in the vehicle control (0.0% ATB1651 gel) and T4 (3.0% ATB1651 gel) treatment groups during the recovery period

No significant change in minipig body weight was observed after 4 weeks of dermal administration or during the 2-week recovery period (Fig. 1). A 1.0% decrease in the mean body weight was observed in 2/5 female minipigs in the control group after 2 weeks of dermal administration; however, the decrease was not statistically significant.

#### Clinical observation and dermal scores

The number of minipigs presenting with clinical symptoms related to ATB1651 gel administration at the application sites is shown in Table 1. Clinical symptoms (including raise spots, erythema and eschar formation, scab, and edema) were observed at ATB1651 gel application sites in males in the 3.0% group and in females treated with formulations containing ≥ 1.0% ATB1651 gel (Table 2). Specifically, raise spots were observed at application sites in both sexes (males: 3/5, females: 3/5) in the 3.0% group day 11–17 of treatment. Scabs were observed only in males and females in the 3.0% group from day 12 to the day of necropsy. Additionally, erythema and eschar formation were observed at the application site(s) in both sexes in the 3.0% group (males: 4/5, females: 3/5) and in 1 female in the 1.0% group. The severity of erythema and eschar with repeated dose administration was mild or well-defined in both sexes from day 11 and persisted until the day of necropsy. Mild or moderate edema was observed at the application site(s) in 2 males and females in the 3.0% group, persisting from day 12 and from days 14–18 in males and females, respectively, to the day of necropsy.

**Table 1:** The number of minipigs with clinical observations related to ATB1651 gel administration at the application site(s) for 4 weeks and the 2-week recovery period

Sex	Treatment				Recovery	
	Male		Female		Male	Female
Group	T3 (1.0%)	T4 (3.0%)	T3 (1.0%)	T4 (3.0%)	T3 (1.0%)	T4 (3.0%)
Total number of animals	3	5	3	5	3	5
Erythema and eschar formation	–	4	1	3	–	1
Edema	–	2	–	2	–	–
Raise spots	–	3	–	3	–	–
Scab	–	1	–	1	–	–

**Table 2:**
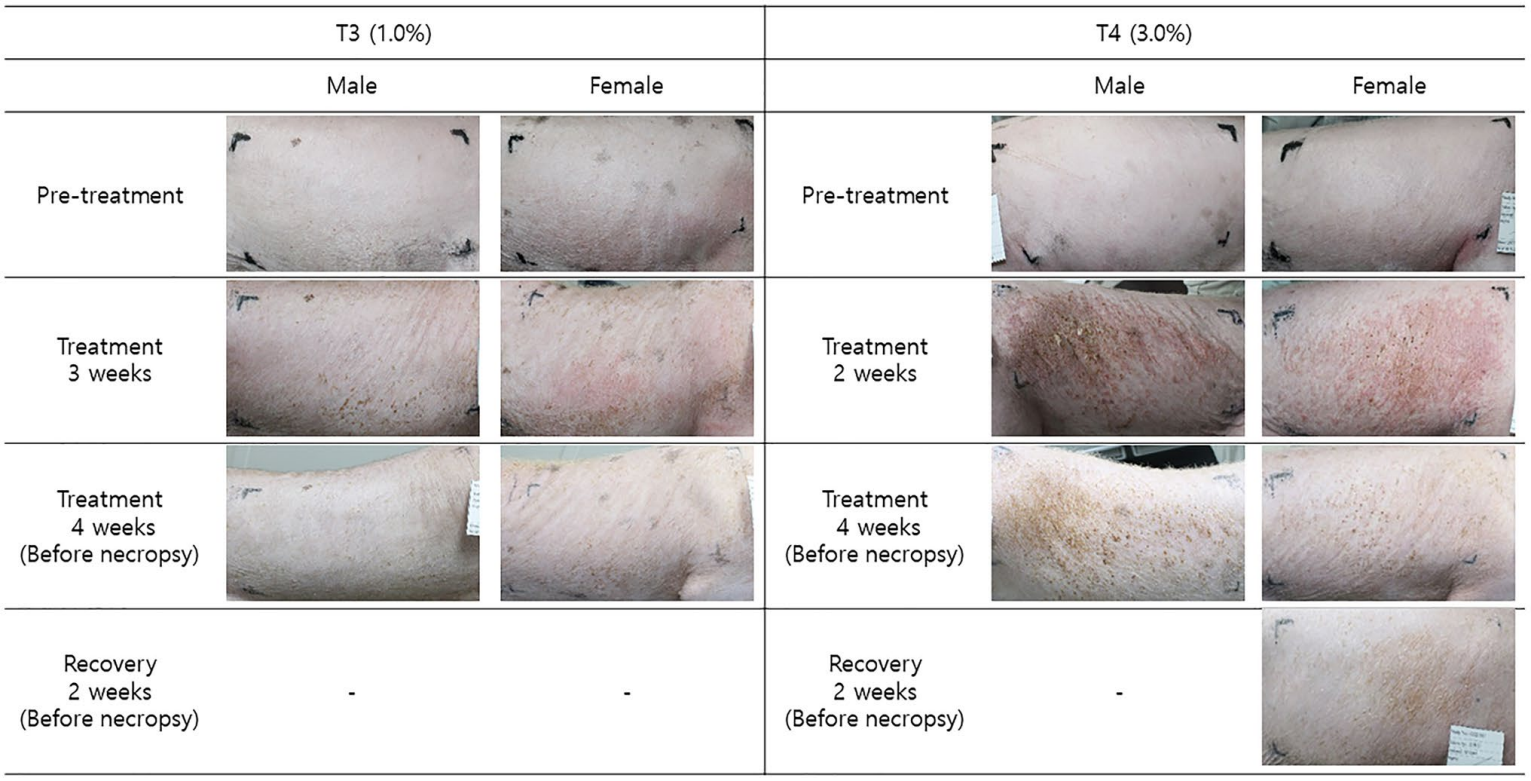
Representative images at the application site(s) following 4 weeks of ATB1651 gel dermal administration

#### Dermal scores

Dermal scores were assigned based on the severity of erythema, eschar, and edema at the application site(s) (Table 3). Males in the 3.0% group and females treated with formulations containing ≥ 1.0% of ATB1651 had relatively high dermal scores (Table 3), which were associated with the clinical symptoms of skin reactions. The onset of dose-dependent erythema/eschar at the application site(s) was observed after 15 days of treatment. The incidence and severity of dermal reactions were highest (T2–T4: grade 2) in both male and female minipigs treated with formulations containing ≥ 0.5% ATB1651 gel between day 15 and 22, which began to decrease from day 23 to 29. Dermal scoring was not performed until the end of the administration. Edema was observed at the application site(s) on days 15 and 22 only. Bare perceptible (grade 1) dermal reactions were observed only in males in the 3.0% group.

**Table 3:** The number of minipigs with erythema and eschar and edema at the application site(s) following 4 weeks of ATB1651 gel dermal administration

	Treatment						Recovery	
	Day 15		Day 22		Day 29		Day 15	
Group	T3 (1.0%)	T4 (3.0%)	T3 (1.0%)	T4 (3.0%)	T3 (1.0%)	T4 (3.0%)	T3 (1.0%)	T4 (3.0%)
Erythema & Eschar								
Male								
Grade 1	–	1	–	2	–	–	–	–
Grade 2	–	2	–	2	–	2	–	–
Female								
Grade 1	–	2	–	–	–	1	–	–
Grade 2	–	1	1	3	–	–	–	–
Edema								
Male								
Grade 1	–	1	–	1	–	–	–	–
Grade 2	–	–	–	–	–	–	–	–
Female								
Grade 1	–	1	–	–	–	–	–	–
Grade 2	–	–	–	–	–	–	–	–

#### Food consumption

No significant differences in the food consumption of both sexes following dermal application of the formulations were observed between the control and experimental groups.

#### Ophthalmology and electrocardiography examination

No significant differences in ophthalmological or electrocardiographic parameters (in both males and females) following dermal application of ATB1651 gel at all concentrations were observed between the control and treatment groups.

#### Clinical pathology

Dermal application of ATB1651 gel at all concentrations did not significantly affect the hematological, biochemical, and urine parameters of the minipigs regardless of sex or group. No significant dose-dependent relationship was found.

### Observations and measurements after necropsy

#### Macroscopic examination

Macroscopic examination after necropsy indicated the presence of mild to moderate scabs in males and females treated with formulations containing ≥ 0.5% and ≥ 0.2% ATB1651 gel, respectively. After the recovery period, scabs with minimal severity were observed in both sexes in the 3.0% group. The macroscopic findings are summarized in Table 4. All other macroscopic findings outside the application site(s), such as scratches or scabs, were considered incidental and are usually associated with cage-housed minipigs.

**Table 4:** The number of minipigs with macroscopic observations at the application site(s) following 4 weeks of ATB1651 gel dermal administration

Sex	Males					Females				
Group	VC (0%)	T1 (0.2%)	T2 (0.5%)	T3 (1.0%)	T4 (3.0%)	VC (0%)	T1 (0.2%)	T2 (0.5%)	T3 (1.0%)	T4 (3.0%)
Number of Animals	3^a^ (2)^b^	3	3	3	3^a^ (2)^b^	3^a^ (2)^b^	3	3	3	3^a^ (2)^b^
Scab at application site(s)	0 (0)	0	3	2	3 (1)	2 (0)	1	2	1	3 (1)

^a^Animal counts in main groups

^b^Animal counts in recovery groups

#### Organ weight

Organ weights (mean ± standard deviation) are presented in Table 5. Following dermal application of ATB1651 gel at all concentrations, no significant differences in organ weights (absolute and relative weights) were observed between the control group and treatment groups.

**Table 5:** The number of minipigs with microscopic observations at the application site(s) following 4 weeks of ATB1651 gel dermal administration

Sex	Males					Females				
Group	VC (0%)	T1 (0.2%)	T2 (0.5%)	T3 (1.0%)	T4 (3.0%)	VC (0%)	T1 (0.2%)	T2 (0.5%)	T3 (1.0%)	T4 (3.0%)
Number of Animals	3^a^ (2)^b^	3	3	3	3^a^ (2)^b^	3^a^ (2)^b^	3	3	3	3^a^ (2)^b^
Application site(s)										
Infiltration, mixed cell	0 (0)	0	1	2	2 (0)	0 (1)	0	0	1	3 (1)
Pustule/crust	0 (0)	0	0	1	3 (1)	0 (1)	0	0	0	3 (1)

^a^Animal counts in main groups

^b^Animal counts in recovery groups

#### Histological changes

**Fig. 2:**
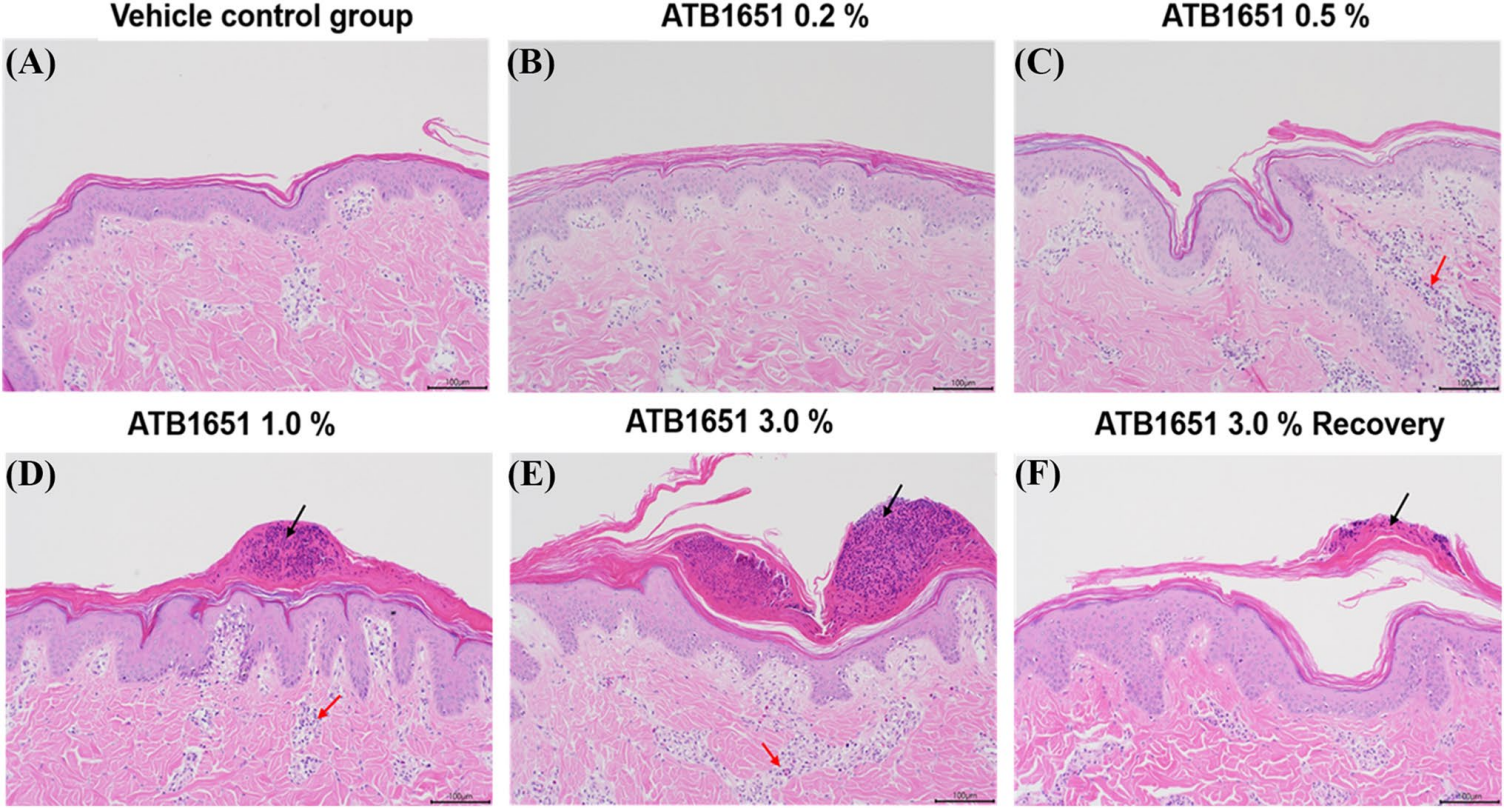
Representative images of hematoxylin and eosin (H&E) staining of application sites after dermal administration of ATB1651 gel for 4 weeks. **a** vehicle control group (× 200), **b** ATB1651 gel 0.2% treatment group (× 200), **c** ATB1651 gel 0.5% treatment group (× 200), **d** ATB1651 gel 1.0% treatment group (× 200), **e** ATB1651 gel 3.0% treatment group (× 200), and **f** ATB1651 gel 3.0% treatment group (× 200) after recovery period. Scale bar = 100 μm. Black arrows, pustule/crust; red arrows, mixed cell infiltration

Histological changes in minipigs following dermal administration of ATB1651 gel for 4 weeks and the 2-week recovery period are shown in Table 5. A dose-dependent mild to moderate inflammatory cell infiltration into the dermis and epidermal-dermal junction layer was observed in males and females treated with formulations containing ≥ 0.5% and ≥ 1.0% ATB1651 gel, respectively. Specifically, the inflammatory cells include eosinophils, monocytes, and lymphocytes (Fig. 2). However, these changes reversed after the recovery period and were associated with inflammatory processes induced by the formulations. Additionally, pustule/crust characterized by focal accumulation of degenerated neutrophils, cellular debris, and eosinophilic material were observed within the stratum corneum of the epidermis in males and females treated with ATB1651 gel formulations ≥ 1.0% and 3.0%, respectively, which was dose dependent (Fig. 2). However, the histological changes reversed after the recovery period and were associated with the local irritation effect induced by the formulations. All other microscopic findings were considered spontaneous or incidental changes commonly observed in minipigs [29].

### Toxicokinetics

**Fig. 3:**
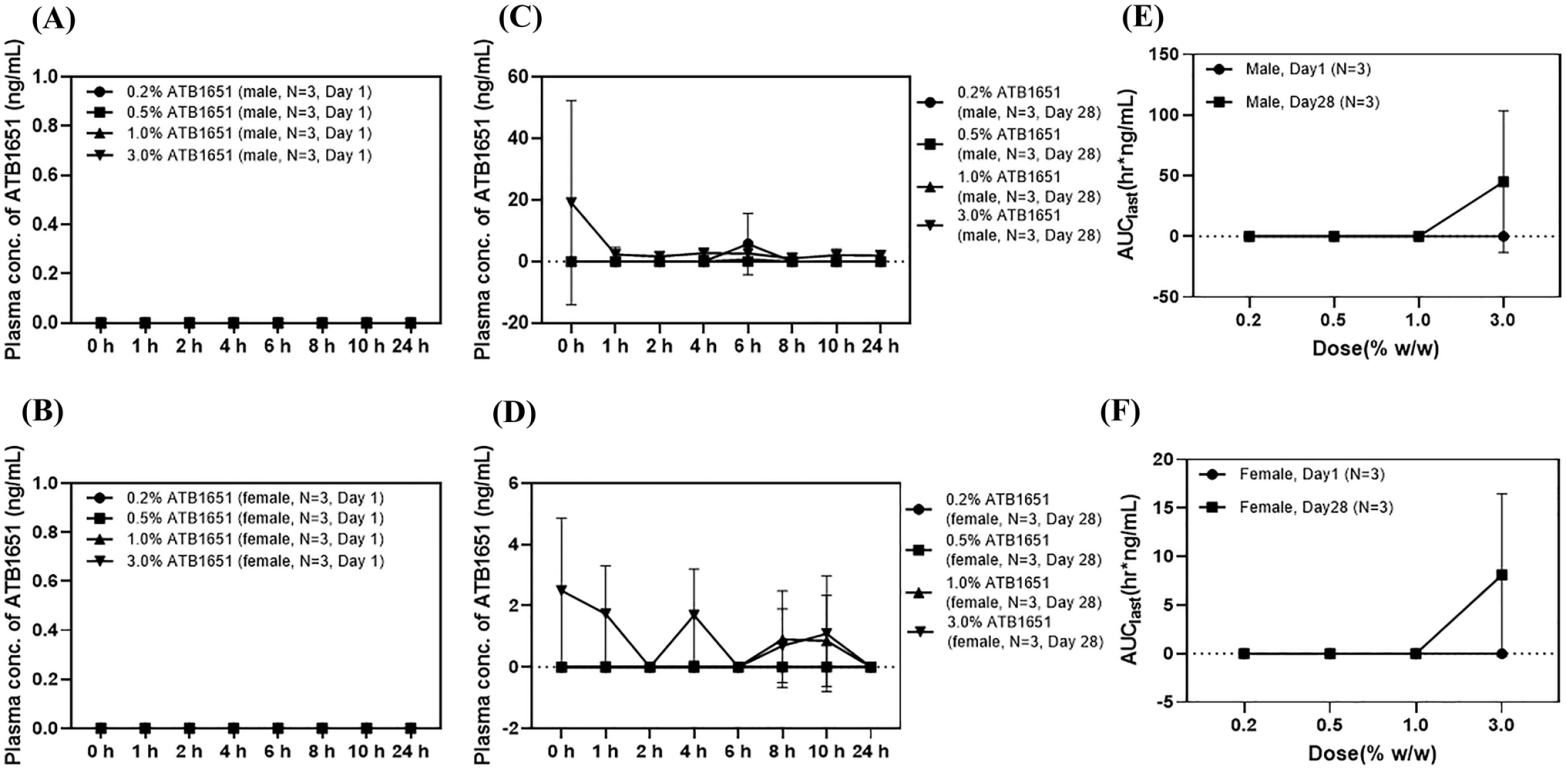
Graph of plasma concentrations (mean ± standard deviation) of minipigs receiving 4-week-repeated-doses of ATB1651 gel via dermal administration. Mean plasma concentration changes in males (**a**) and females (**b**) of ATB1651 gel treatment groups on day 1 in males (**c**), and females (**d**) of ATB1651 gel treatment groups on day 28. AUC_last_ indicates the comparison between day 1 and day 28 in males (**e**) and females (**f**)

Plasma concentrations of minipigs administered dermally with ATB1651 gel at 0.2, 0.5, 1.0, and 3.0% on days 1 and 28 are shown in Fig. 3. ATB1651 gel systemic exposure (AUC_last_) was evaluated on day 28 in only 4 minipigs in the 3.0% group because the amount of ATB1651 gel was below the limit of quantification (BLQ) or undetectable in the plasma of minipigs in the other groups. The AUC_last_ values were 12.17 h ng/mL and 67.60 h ng/mL in males and females in the 3.0% group, respectively. Additionally, a dose increase from 1.0 to 3.0% increased the C_max_ for ATB1651 by 1:1.46 and 1: 10.67 in females and males, respectively.

The unusually high C_max_ in males in the 3.0% group was due to a significantly higher ATB1651 gel concentration in pre-dose samples from 1 male compared with that of other samples in the same group. The AUC_last_ for ATB1651 gel was 18.0% higher in females in the 3.0% group than in males in the same group on day 28. The effect of sex on the toxicokinetics of ATB1651 gel could not be evaluated in this study due to insufficient data.

## Discussion

Antifungals are effective in treating nail infections and candidiasis by suppressing the production of ergosterol, a component of fungal cell membranes and inhibiting cell membrane permeability [3, 4, 30]. Currently, antifungal agents are administered systemically (oral or intravenous) or topically (spray, cream, or gel) to treat fungal infections. However, toxicity has been reported in these routes of drug administration.

In human clinical studies, systemic administration of polyenes, such as amphotericin B and Terbinafine, has been associated with renal, gastrointestinal, and cardiac toxicity, subcutaneous tissue disorder, general disorders, and erythema [31–36]. Additionally, azole systemic administration has been reported to cause hepatotoxicity [12, 37]. Moreover, nervous system and general disorders are associated with voriconazole and isavuconazole administration, whereas gastrointestinal and cardiac disorders are associated with itraconazole and posaconazole, respectively [35, 38]. Furthermore, systemic administration of echinocandins has been reported to induce hepatotoxicity, nephrotoxicity, and skin and subcutaneous tissue disorders [35, 38]. In veterinary clinical studies [39], polyene (amphotericin B) systemic administration has been reported to cause nephrotoxicity via vasoconstriction, impaired acid excretion, and direct tubular injury in dogs [40, 41], in addition to nephrotoxicity in rats and mice [42–45]. However, topical antifungal drugs have proven effective for treating various fungal infections without systemic adverse effects. Minipig skin has immunological, anatomical, and physiological similarities with human skin; thus, the use of minpigs in dermal toxicity studies has increased in recent years [18, 20, 22]. In addition, the skin of pigs has similar characteristics with that of humans, including firm attachment to the subcutaneous tissue, sparse hair coat, thick epidermis and dermis, similar dermal epidermal thickness ratio, absence of panniculus carnosus, epidermal turnover time, re-epithelialization as a healing mechanism, vascular anatomy, and collagen structure [46, 47]. However, pig skin has apocrine glands, whereas human skin has sweat glands [47]. Furthermore, pig skin has a slightly higher pH (6–7) than human skin (5) [21]. Nevertheless, dermal evaluation using minipigs has been reported as a more accurate evaluator of dermal toxicity than that using rodents because the dense hair follicles of rodents lead to a high transdermal absorption rate [48].

Based on skin characteristics, minipigs are highly accepted as an alternative to rodents for dermal studies in Europe and the USA, which has also been suggested in guidelines from Japan and Canada. The US EPA and OECD Test Guidelines 409 and 410 (https://www.oecd-ilibrary.org/environment/test-no-410-repeated-dose-dermal-toxicity-21-28-day-study_9789264070745-en) listed swine and minipigs as optional species for dermal studies. Therefore, minipigs are primarily used for dermal toxicity and efficacy studies of candidate topical drugs. Particularly, information on topical antifungal drug toxicity in minipigs has been provided by the NDA [49, 50].

In the present study, we investigated the potential dermal toxicity of ATB1651 gel application at different concentrations in Yucatan minipigs. Specifically, the formulations were applied to 10% BSA for ~ 6 h daily for 4 weeks. Histopathological assay showed a dose-dependent infiltration of different inflammatory cells, and the presence of pustule, crust, and scabs in males and females treated with formulations containing ≥ 0.5% and ≥ 1.0% ATB1651 gel. However, these histopathological changes generally returned to normal after the 2-week recovery period. Clinical symptoms included redness, scabs, and raised spots, which were considered to be local irritation effects of ATB1651 gel. However, other parameters, including body and organ weight and hematological, biochemical, and urine parameters, were not significantly affected by the antifungal gel. Additionally, ATB1651 was detected only in the plasma of minipigs in the 3.0% group, thus indicating that systemic exposure after day 28 and the treatment induced considerable changes at the application sites. However, there were no systemic changes, such as body weight, organ weight, or pathological changes. Consequently, an ATB1651 gel concentration of 3.0% was determined as the NOAEL.

In our own preliminary study, the effective dose was 0.5% w/w when ATB1651 was administered dermally to the skin of rats intradermally infected with fungus; thus, the systemic and topical NOAEL in the present toxicity study were above the effective dose. The therapeutic window is defined as the dose range between the minimum effective concentration and the minimum toxic concentration [51]; thus, the therapeutic window in this study is 0.5–3.0% w/w. As the 3% w/w concentration is not toxic and is the NOAEL, the therapeutic window is expected to widen. Therefore, ATB1651 can be considered safe for clinical development as an antifungal drug with a wide therapeutic window.

Conclusively, the findings of this study showed that ATB1651 gel-induced dermal alterations were limited to the application site(s) and reversible during the recovery period. Moreover, these dermal alterations were considered to be related to the local irritation effect of the formulations. Therefore, the NOAEL of ATB1651 gel was considered to be 3.0% for topical and systemic toxicity in both male and female minipigs.

## Data Availability

All data needed to evaluate the conclusions presented in this study are present in the paper and the Supplementary Materials. Additional data related to this paper may be requested from the authors.
